# The Politics and Crisis Management of Animal Health Security

**DOI:** 10.3201/eid2112.151507

**Published:** 2015-12

**Authors:** Jennifer McQuiston

**Affiliations:** Centers for Disease Control and Prevention, Atlanta, Georgia, USA

**Keywords:** animal health security, politics, crisis management, zoonoses, foot and mouth disease, United Kingdom, picornavirus, viruses

Even as the lessons learned from the response to the 2014–2015 Ebola outbreak in West Africa begin to be analyzed, history provides other key examples of health crises that challenge traditional thinking and, in some cases, government structures themselves. In his new book The Politics and Crisis Management of Animal Health Security, author John Connolly examines the events and aftermath of the 2001 foot and mouth disease (FMD) outbreak in the United Kingdom, an event that cost >£8 billion (US ≈$11.6 billion), resulted in the death or slaughter of 6.5 million animals, and led to the country’s first delay in a national election since World War II.

Caused by a picornavirus, FMD affects cloven-hoofed animals including sheep, cattle, and pigs. Because of the disease’s highly infectious nature, FMD-free countries like the UK must respond quickly to outbreaks because of economic trade consequences; a quick and well-coordinated response is key to success. However, in 2001, some early and notable missteps in UK government decisions, including a 3-day lag in imposing national animal movement bans, led to viral spread via animal movements and a mushrooming of the national crisis. The atmosphere of the outbreak response was highly charged, and battles between government and stakeholders played out daily in the media with political cartoons and images of burning carcasses. The crisis led to the dissolution of the country’s Ministry of Agriculture, Forestry and Fisheries and the creation of the new Department for Environment, Food & Rural Affairs (DEFRA). In his book, Connelly asks, have the lessons learned during the aftermath of the 2001 FMD outbreak been adequately applied, and is the UK better prepared to deal with a substantial animal health crisis in the future?

Written in multiple parts, this book provides an initial primer in crisis management and defines theoretical drivers of change. Connelly describes the FMD outbreak in the United Kingdom as a “critical juncture” situation: that is, a public crisis combined with the political climate to drive necessary organizational change. Next, the book takes the reader through a description of the acute crisis, including technical details of the outbreak’s spread and the primary features of the government response. The book then moves the reader into an understanding of DEFRA’s post-FMD crisis management and how the new agency tackled a “fix” to the problem through contingency planning, improved communications, science advocacy, and stakeholder engagement. Finally, the book reviews the efficacy of DEFRA’s new plans, as executed during a 2006–2007 avian influenza A(H5N1) crisis and a 2007 outbreak of FMD associated with an accidental laboratory release. The author’s conclusions are that many of the lessons learned from the 2001 outbreak have resulted in positive change and a stronger capacity for the government to respond to recent and future outbreaks.

Reviewing this book carries personal significance for me, as I spent a month in England in 2001 helping the United Kingdom as part of the response. I talked directly with the farmers at the epicenter of the outbreak and saw firsthand the public distrust fueled by challenges in communicating government decisions. Now, working in public affairs and scientific communications myself, I found it very informative to review this book and look back at that time through a new professional lens. The book lacks, perhaps, a robust discussion of the role of media and public outcry in shaping government policy decisions, but nonetheless proves an excellent reference for anyone who wishes to understand organizational change in response to crises, particularly in the health and veterinary sectors. The positive changes that have taken place since 2001 provide me with hope that the United Kingdom—and other countries that choose to pay close attention—may indeed be better prepared for future outbreak responses.

